# Evaluation and classification of obfuscated Android malware through deep learning using ensemble voting mechanism

**DOI:** 10.1038/s41598-023-30028-w

**Published:** 2023-02-22

**Authors:** Sana Aurangzeb, Muhammad Aleem

**Affiliations:** Department of Computer Science, National University of Computer and Emerging Sciences (FAST-NUCES), Islamabad, 44000 Pakistan

**Keywords:** Computer science, Data mining, Machine learning

## Abstract

With the rise in popularity and usage of Android operating systems, malicious applications are targeted by applying innovative ways and techniques. Today, malware becomes intelligent that uses several ways of obfuscation techniques to hide its functionality and evade anti-malware engines. For mainstream smartphone users, Android malware poses a severe security danger. An obfuscation approach, however, can produce malware versions that can evade current detection strategies and dramatically lower the detection accuracy. Attempting to identify Android malware obfuscation variations, this paper proposes an approach to address the challenges and issues related to the classification and detection of malicious obfuscated variants. The employed detection and classification scheme uses both static and dynamic analysis using an ensemble voting mechanism. Moreover, this study demonstrates that a small subset of features performs consistently well when they are derived from the basic malware (non-obfuscated), however, after applying a novel feature-based obfuscation approach, the study shows a drastic change indicating the relative importance of these features in obfuscating benign and malware applications. For this purpose, we present a fast, scalable, and accurate mechanism for obfuscated Android malware detection based on the Deep learning algorithm using real and emulator-based platforms. The experiments show that the proposed model detects malware effectively and accurately along with the identification of features that are usually obfuscated by malware attackers.

## Introduction

Android Operating System (OS), having more than two million applications continues to grow in such a scalable and large competitive environment^1^. To compete efficiently and effectively, malicious cyber threats are required to be detected using a reliable mechanism.

Related to malware detection, the three most common challenges are: to identify obfuscated malware, classify them, and identify the important features useful in the application obfuscating. This investigation requires a deep analysis of the potentially dangerous applications^2^, therefore requiring a lightweight mechanism to cope with such a huge number of applications. Obfuscation of malware usually can be applied through several ways such as string encryption-based obfuscation, junk code insertion^3,4^, using fake try-catch statements^5^, repacking application^6,7^, call indirection^8^, code re-ordering^9^, package renaming^10^, native code obfuscation^11^, dynamic code loading^12^, code hiding^13^, reflection, etc. The variety and heterogeneous nature of the potential obfuscation techniques make them extremely difficult to identify.

Generally, malware analysis is performed using three different approaches i.e., static, dynamic, and hybrid analyses^14^. Static-based analyses are computationally less expensive and lightweight, however, these approaches are easily thwarted by the obfuscation mechanisms^15^. Using dynamic-based analyses, obfuscated malware could be effectively detected, however, generally, these approaches require more technical expertise and are computationally expensive^16^. The third approach i.e., hybrid-based analyses employs both the static and dynamic-based investigations in such a way as to efficiently utilizes the advantages of both approaches to overcome their potential shortcomings^17^. However, the hybrid approach is mostly more costly. In the literature, there are multiple techniques that have proposed lightweight features suitable for applying Machine Learning (ML) based classification^15^. Despite the promising results of using these features with ML models, the usage of these aspects for the classification of the obfuscated malware is a challenging task^2^. In this paper, we intend to address the above-mentioned challenges. To analyze potentially dangerous apps (i.e., obfuscated malware), we propose an Android malware classification approach that utilizes features extracted from a comprehensive malware dataset *Kronodroid*^18^, that contains a vast range of malware collected from the year 2008 to 2020. The proposed approach aims to cater to obfuscation intelligently through Deep Learning (DL) as well as traditional machine learning algorithms using an ensemble voting mechanism. The experimental evaluation shows promising results that the proposed approach is lightweight and effective to detect and classify obfuscated malware.

The rest of the paper is organized as follows. Section 2 highlights the motivation and contributions of this work, Section 3 presents a detailed literature review along with its summary. Section 4 presents the proposed approach, feature selection process, feature extraction mechanism, and evaluation measurements. Section 5 provides results and related discussion. Section 6 concludes the paper and highlights the future directions.

## Motivation and contributions

The Android application, sponsored by Google, comes up as an archive i.e., .apk file. The .apk file is a compressed package file, however, when we decompress it the application includes several files and folders such as manifext file, delvic bytecode i.e., classes.dex file, native libraries, resource folder, signatures i.e., META-INF folder. These files contain the functionality of Android applications as well as all the resources used, symbols, layout information, application version specifications, name of packages used, code and operating system instructions, etc. Obfuscating an application means changing the code in such a way that the functionality remains the same while the code becomes very challenging to understand. Therefore, obfuscation-based malware are more tends to evade the detection system. Obfuscating a file mainly includes string encryption, code insertion, variable renaming, package renaming, class name change, resource obfuscation, intents obfuscation, inserting random code, API obfuscation, and permission obfuscation, etc. Applications that adapt obfuscation are of two purposes: (i) to obfuscate malware applications in order to hide malicious content that becomes difficult to read, thus, evading detection mechanism; (ii) to obfuscate benign applications to protect code privacy or intellectual property. Both the malicious and benign applications are obfuscated which becomes difficult to classify. Therefore, in this study, we aim to identify the benign and obfuscated malware to classify them correctly by identifying those features that play a crucial role in identifying obfuscated behavior. Moreover, to analyze critical malware apps (i.e., the obfuscated malware), we employed the dataset named *Kronodroid*, which contains a vast range of malware collected from the year 2008 to 2020. The Kronodroid dataset contains the most recent and advanced malware samples (i.e., obfuscated apps). The main motivation for choosing this as compared to the traditionally used data sets (e.g., Drebin^19^, Malgenome^20^, AndroZoo, etc.) is that the traditional data sets are mainly used for the classification of malware and benign applications and lack a good number of advanced malware such as those malware that employs several encryption techniques (e.g., polymorphic behavior). With technological advancements, the traditional malware represents less sophisticated and older malware samples and is not adequate for the analysis of the newer malware threats based on obfuscation^18^. Also, the dataset size in terms of the number of samples is smaller as compared to the employed Kronodroid dataset. For example, the MalGenome dataset contains 1260 samples whereas, the Drebin dataset consists of 5560 samples from the year 2010 to 2012. In contrast, the dataset employed in this study (i.e., Kronodroid) contains more diversified malware samples i.e., the older ones with new and advanced samples from the year 2008 to 2020. The number of samples suitable for the controlled environment experimentation consisted of 63,991 samples while 78,137 are suited for the real environment or Android devices-based experimentation.

Keeping the importance of Android malware classification in mind, we make the following contributions to the state-of-the-art approaches:

1. We proposed an ensemble voting-based approach capable of effectively classifying Android applications potentially augmented with obfuscation;
2. A novel features-based obfuscation mechanism is applied to identify the crucial attributes contributing to obfuscated malware;
3. A comprehensive malware dataset i.e., *Kronodroid* is employed containing the initial Android-based malware to the recent ones;
4. A extensive evaluation is performed using both the *real Android device* and an *Emulator-based* platforms;

## Related work

In Suarez-Tangil et al.^2^ authors proposed a static-based approach: DroidSieve, an Android-based malware detector to decide whether the app is malicious or not. If the first statement is true it classifies the app further to identify the particular family of malware. The DroidSieve approach utilizes features including API calls, code structure, permissions, and the set of invoked components to handle obfuscation. For the malware detection, the authors achieved 99.82% accuracy with zero false positives whereas, for family identification of the obfuscated malware, authors attained 99.26% accuracy. However, the proposed approach does not employ dynamic analysis resulting in the evasion of the malware having run-time obfuscation. The amount of the dataset used for the experimentation is adequate, however, the employed dataset contains the apps which are older and outdated (well-known malware).

In Sihag et al.^21^ authors presented BLADE, an obfuscation resilient mechanism based on Opcode Segments for the detection of malware and feature characterization. The authors also, performed semantics-based simplification of the Dalvik opcodes to enhance their resilience. To evaluate the effectiveness of the proposed approach against a benchmark dataset for malware detection, and family classification. Different obfuscation techniques such as trivial obfuscation, string encryption, class encryption, reflection, and their combinations are used. However, obfuscation techniques against the native code, libraries, and code if placed outside the DEX file are ignored. Experiments were conducted in a controlled environment. Moreover, dynamic analysis is not considered as a result of which sophisticated malware attacks are ignored. In addition, dynamic analysis of obfuscated malware samples is not considered.

Lee et al.^22^ proposed to detect obfuscated malicious Android applications, authors have applied stacked Recurrent Neural Networks (RNN) and Convolutional Neural Networks (CNN) to identify malware by learning obfuscated string patterns from an application’s package name and the certificate owner name. Their experiments demonstrate that the proposed approach outperforms Ngram-based models and is robust to obfuscation for Android devices. However, the dataset used in this study is older than 2017 that lacks sophisticated and obfuscated samples of the latest malware. Moreover, dynamic analysis is not considered as a result of which obfuscated malware samples mostly evade.

In Wu et al.^23^ authors proposed and designed IFDroid: a system to achieve robust classification methods to address the issues of code obfuscation problems. The features obtained through program analysis contain many useless and disguised features that result in false negative indications. Therefore, the authors identify the obfuscation-resilient malware analysis through contrastive learning that can be used to reduce the difference produced by obfuscation. In addition, authors have transformed the function call graph of a sample into an image by centrality analysis. Later, the authors generated heatmaps that were obtained through visualization techniques for family classification. However, the study ignored several obfuscation techniques such as dynamic-based obfuscation.

In^24^ Tang et al. proposed an anti-obfuscation Android malware detection system known as MGOPDroid. The proposed approach extracts opcode features with different granularities and then combines the TF-IDF algorithm with the difference-index of opcode feature distribution before and after applying obfuscation to calculate the weight of the opcode features. Later, these opcode sequences are converted into grayscale images to achieve features’ visualization. The authors have employed a deep learning detection model combined with image enhancement, Resnet, and global average pooling layer to detect malware variants. The reported results indicate that the malware detection accuracy for un-obfuscated samples is 96.35%, and 94.55% for the obfuscated malware. For malware family classification, the accuracy is 95.31%, whereas, after obfuscating, the classification accuracy rate is 89.96%. However, the achieved accuracy is from static-based obfuscation whereas, the applications with dynamic code loading are not considered. The dynamic-based obfuscation is crucial considering the advanced malware emerged recently.

In^25^ Jusoh et al. have presented a survey and discussed the articles published from the year 2009 to 2019 to analyze the procedure involved during the static analysis (i.e., reverse engineering, feature extraction, and classification) with taxonomy. Furthermore, the authors have indicated that many researchers and practitioners are using API and manifest files which have great significance. Moreover, the authors concluded that the classification could be done using traditional machine learning, deep learning, graph, and other suitable approaches to enhance the malware classification accuracy.

Table 1 presents the related work summary.

**Table 1:** Summary of the proposed approach with the state-of-the-art existing approaches.

Paper	Methodology	Strengths	Weaknesses
Suarez-Tangil et al.^2^	Static-based approach Features: API calls, code structure, permissions, set of invoked components Dataset used are: Malgenome Project and the Drebin dataset	Family Identification Analyzed reflection and encryption techniques Classifier used: Extra Trees identify obfuscated malware Achieved 99% accuracy	Dynamic analysis not considered Sophisticated malware not considered Dynamic obfuscated malware samples not considered Outdated dataset used Controlled environment i.e., emulator
Sihag et al.^21^	Feature: opcode for obfuscation Datasets used are: Androtopsy, AndroTracker, Drebin and Android PRAGuard	Family identification Opcode Segments for detection Trivial obfuscation, string encryption, class encryption, reflection and their combinations are used Classifiers used: k-NN, J48, RF and SMO Achieved 92.47% accuracy	Obfuscation technique against native code, libraries ignore Deep learning algorithms not used Controlled environment i.e., emulator Dynamic analysis not considered Sophisticated malware ignored Dynamic obfuscated malware samples not considered
Lee at al.^22^	Features: using Ngrams from package and certificate owner strings Dataset used: VirusTotal	Using Android metadata for classification Classifiers used: CNN and RNN	Dataset used is older than 2017 Dynamic analysis not considered Sophisticated malware are ignored Dynamic obfuscated malware samples not considered Several obfuscation technique are ignored Controlled environment i.e., emulator
Wu et al.^23^	Static-based approach Obfuscation-resilient analysis achieved via contrastive learning Datasets used: MalGenome and AndroZoo	Family identification Transformed the function call graph of a sample into an image by centrality analysis Heatmap visualization technique is used for family identification Achieved 98.4% accuracy	Dynamic analysis not considered Updated malware ignored Dynamic analysis of obfuscated malware samples not considered Several obfuscation technique are ignored Experiments conducted in controlled environment
Tang et al.^24^	Android malware detection based on multi-granular opcode features Dataset used: Drebin Obfuscation applied using AVPass tool Adopted a feature weight calculation method combining TFIDF	Image-based detection and classification is conducted The difference in opcode features before and after applying obfuscation is performed Achieved 95.31% accuracy without obfuscation and 89.96% acheived after applying obfuscation Deep learning classifiers are used	Lack of sophisticated malware dataset Image conversion and generation takes time System does not considered the analysis of packed Android applications Dynamic applications withdynamic code loading is not considered.

From the literature, it can be seen that most of the studies are conducted in a controlled environment where sophisticated malware tends to hide its functionalities by applying different obfuscation techniques. Whereas in this study we perform the comparison using both types of environment i.e, real and emulator devices. Furthermore, several studies used the datasets either from Drebin^19^, or Malgenome^20^, or AndroZoo, etc., which are mostly used for benign and malware classification purposes. However, these datasets lack a good number of advanced malware such as those malware that employs several encryption techniques such as polymorphic. Because of technological advances, malware becomes sophisticated, making these datasets to be outdated or old in terms of malware advancements^18^. In addition, the samples from these datasets are relatively small, especially in the case of MalGenome which is composed of just 1260 samples whereas, Drebin consists of 5560 samples from the year 2010 to 2012. However, the dataset used in this study is a combination of old as well as recent malware from the year 2008 to 2020 consisting of 63,991 samples suitable for controlled environment experimentations while 78,137 for the real environment or Android devices. Similarly, there are several studies that are based on static analysis to achieve high accuracy and quick detection, however, these approaches lack detection of the advanced malware that employs dynamic obfuscation^24^. However, this study considers the shortcomings of both approaches i.e., static and dynamic analysis by utilizing a deep learning ensemble voting mechanism for effective detection and classification of advanced malware. In addition, several existing studies use traditional machine learning as compared to the deep learning-based mechanisms which result in less effective detection in the case of obfuscated malware analyses considering application behavior^25^. Moreover, it is very valuable to highlight specific features which have a significant role in application obfuscation.

## The proposed approach

**Figure 1:**
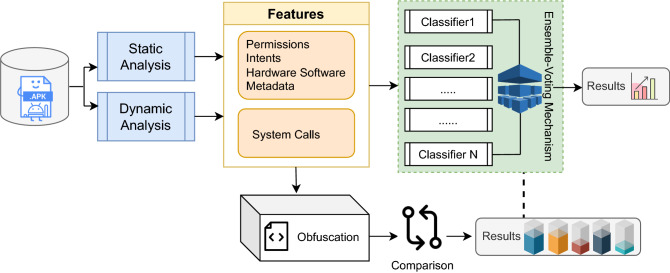
Proposed approach methodology.

The proposed approach utilizes the aspects of both static and dynamic analysis to evaluate Android applications that may be malware or benign application. For the evaluation, we use the dataset *Kronodroid*, which is also termed as a hybrid dataset (see section *Dataset* for details). The features extracted after static analysis are *Permissions*, *Intents*, *Hardware* and *Software*, and other metadata. System calls which are also referred to as kernel calls are employed for the dynamic analysis. The features of malware and benign applications are extracted using two devices or platforms: the experiments conducted on real Android devices and the experiments conducted on the emulator (as mentioned in^18^). The features extracted after static and dynamic analysis are then given as input to machine-learning classifiers for three purposes: (i) to correctly classify the applications into malware or benign categories (using machine learning and deep learning classifiers); (ii) to identify the performance and detection rate comparatively among the real device and emulator platform; (iii) to apply feature-based obfuscation to identify the vital attributes that play role in application obfuscation. The proposed approach uses five machine-learning classifiers: Gradient Boosting^26^, KNN^27^, Random Forest^28^, XGBoost^29^, and Multi-layer perceptron (MLP) Neural Network^30^. An ensemble-voting mechanism is then applied to classify the applications as malware or benign based on the majority voting system (as shown in Fig. 1). Combining classifiers is a technique used in ensemble-voting mechanisms and has been widely used in data mining. The literature shows that ensemble voting demonstrates significantly lower error rates in classification problems as compared to using any single ML classifier^31^. Moreover, an ensemble reduces the dispersion of the predictions and model performance^32^.

**Figure 2:**
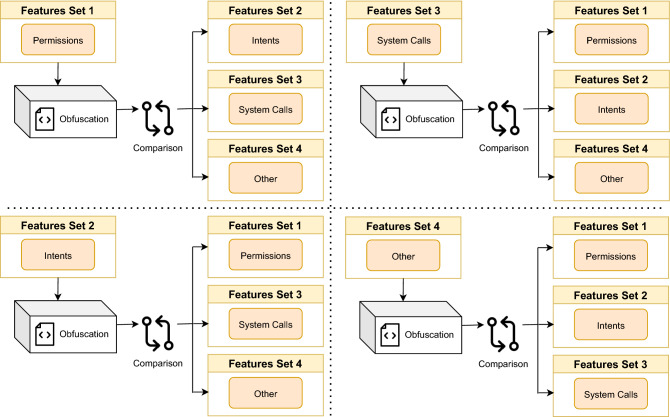
Employment of the feature-obfuscated technique on the proposed approach.

As presented in Figs. 1 and 2, we use feature-obfuscation techniques to modify the features’ values to analyze the impact of the specific artifacts that play a crucial role in the obfuscated applications. As these are the vital attributes that malware attackers usually target to obfuscate and to generate variants that if identified earlier can help in classifying obfuscated malware. After applying *chi-squared*, the features are then divided into 4 feature sections (i.e., system calls, permissions, intents, and the metadata) to comprehensively analyze the obfuscated malware applications. The feature-based obfuscation is applied section by section and results are compared with the other non-obfuscated feature sections to gauge the impact (as shown in Fig. 2). Here, we use the term *feature obfuscation* to represent the process of values modification (of a single category of features as compared to the other features). We have applied *feature-obfuscation* mechanism in such a way that reverting the values arbitrarily from 0 to 1 and 1 to 0 of features having a greater impact in that category and compared it with the other three categories A, B, and C (as shown in Fig. 2). Similarly, we reverted the values of the second category and compared them with the rest of the three categories. These categories are selected after applying *chi-squared* (as explained above). The aim is to observe the change in features that can play a crucial role in malware classification. These results are presented in Table 8, where we can see that after applying feature obfuscation in a controlled environment (i.e., an emulator), there seems slight variation in some of the features whereas no noticeable changes can be seen in other features indicating the greater involvement of these aspects in application obfuscation (see section Results and Discussion for the detailed discussion).

Following are the steps to demonstrate the proposed methodology:

1. Android Apk files are downloaded and unzipped;
2. Static analysis is performed on the emulator as well as the real Android device to get static features (i.e., Permissions, intents, Hardware and Software, Metadata, etc.);
3. Dynamic analysis is performed on the emulator as well as the real Android device to acquire dynamic features (e.g., system calls, etc.);
4. All these features are then used for two purposes;
  - Classification
    i These features are then provided as input to different ML classifiers;
    ii Ensemble learning is applied based on majority voting to decide whether the apk is benign or malware;
  - Applying Feature-Obfuscation
    i feature-obfuscation techniques are now applied on these features to revert the values in order to identify those artifacts that play a crucial role in the application obfuscation
    ii Chi-squared is applied to select the relevant features (i.e., system calls, permissions, intents, and other metadata).
    iii From these 4 feature sections, one section is obfuscated in such a way as to compare the result from the rest of the three sections.
    iv Similarly, one by one the other feature section is reverted to compare the results and to identify the features that played a role in obfuscating malware.
    v Results are then evaluated by the classifiers and demonstrated.

### Dataset

For effective Android malware classification and detection, we used a dataset from KronoDroid^18^, which is a time-based Hybrid-featured Dataset. The reason for using this dataset is because of its robustness and time-based characteristics covering all years of Android history, from 2008 to 2020 including different sources of benign and malware data. In particular, a total of 489 static and dynamic features are collected using an emulator and a real device (as depicted in Table 2). Dynamic features include system or kernel calls whereas static features include permissions, intent filters, and other metadata. The detailed description of the features extracted is mentioned in Table 3.

**Table 2:** Dataset detail.

Device	Benign	Malicious	Total	Dynamic features	Static features
Emulator device dataset	35,256	28,745	63,991	289	200
Real device dataset	36,755	41,382	78,137	289	200

**Table 3:** Particularities of the used dataset.

Hybrid Dataset	Features	Description
Dynamic	System Calls	To analyze the behavioral and runtime features of the application. It provides a level of abstraction and security, acting as handlers o,f service and resource requests from the applications using API (i.e., user-level) to the OS (i.e., kernel-level)
	Permissions	To analyze the security permissions the application needs to access protected resources (i.e., requested permissions)
	Intents	To analyze the actions of the application intended to perform
Static	Hardware and Software	To analyze the required hardware and software features used by the application to perform any of its assigned tasks
	Metadata	To analyze information retrieved from metadata i.e., package name, activities, services declared, broadcast receivers, and content providers, etc.

### Feature extraction, selection, and analysis

Features selection process plays a vital role in machine learning that helps in training the model accurately and effectively. As there are a huge number of features in the dataset and finding the most relevant features is a challenging task. The main objective of the feature selection process is to reduce the number of features by selecting the most relevant ones, and removing the irrelevant and redundant attributes, thus making the model faster to train with the reduced memory footprints. For the feature selection, we have used the chi-squared^33^. The chi-squared approach is used when features are categorical and are used to evaluate the self-determination among two events^34^ with respect to the classes. The Chi-Squared statistics are calculated using the following formula:

$$\begin{aligned} Chi-square = \sum _{i=1}^{\ m} \sum _{j=1}^{\ n} \frac{(O_{ij} - P_{ij})^2}{P_{ij}} \end{aligned}$$

where *m* is the number of intervals, *n* the number of classes, $O_{ij}$ the number of instances of class *j* in the *i*-th interval, “O” stands for observed or actual, and “P” stands for expected value. If these two categories are independent then it means O and P will be close and if they have some association then the Chi-squared value will be high^35^ which indicates a higher value of Chi-squared implies the denial of the null hypothesis and consequently these features can be analyzed as good relevance.

Therefore, using the *chi-squared* algorithm *20* features out of 489 were selected for the emulator device and top *20* features out of 489 were selected for the real device-related dataset. The individual feature sets were then trained on different types of machine learning classifiers with a cross-validation of 10 to check whether the ensemble of classifiers showed a better result. A list of extracted static and dynamic features along with their description is shown in Table 3. For classification purposes considering the nature of the employed data set, we have used four well-known machine learning classifiers: Gradient Boosting, KNN, Random Forest, XGBoost, and one deep learning classifier Multi-layer perceptron Neural Network is used.

### Experimental setup and performance evaluation measurements

We have performed experiments on a stand-alone machine, the specifications are shown in Table 4.

**Table 4:** System configuration.

System	MacBook Pro2019
CPU	2.4GHz 6-Core Intel Core i7
Memory	16 GB 2400 MHz DDR4
GPU	Intel UHD Graphics 630 1536 MB
OS	macOS Big Sur ver 11.0.1
Data mining tool	Python

### Evaluation metrics

For the evaluation of the selected classifiers, we have constructed our findings through accuracy, precision, recall, and F1 Score that is based on TP (True Positive), TN (True Negative), FP (False Positive) and FN (False Negative). Where the TP is all the malware samples recognized correctly as malware, the TN represents all the benign samples recognized correctly as benign, FP is all the benign samples recognized incorrectly as malware and FN is all the malware samples recognized incorrectly as benign. Accuracy is the fraction to which malware and benign samples are recognized correctly. Accuracy, precision-recall, and F1 Score are calculated using the following equations:

1 $$\begin{aligned} Accuracy= & {} \frac{TP + TN}{TP + TN + FP + FN} \end{aligned}$$

2 $$\begin{aligned} Precision= & {} \frac{TP}{TP + FP} \end{aligned}$$

3 $$\begin{aligned} Recall= & {} \frac{TP}{TP + FN} \end{aligned}$$

4 $$\begin{aligned} F1-Score= & {} 2 \times \frac{Precision * Recall}{Precision + Recall} \end{aligned}$$

## Results and discussion

**Figure 3:**
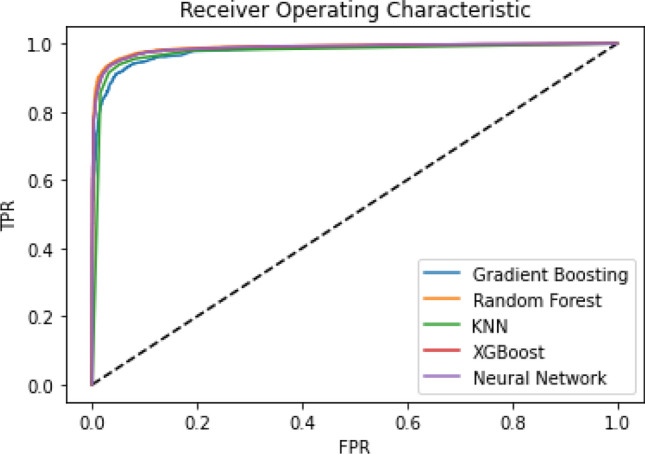
ROC curve comparing Neural Network with other ML classifiers under real device dataset.

**Figure 4:**
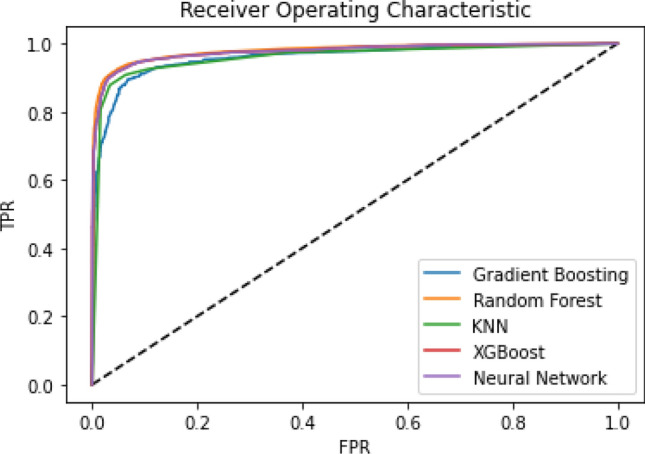
ROC curve comparing Neural Network with other ML classifiers under emulator device dataset.

In this section, we present the results of a wide variety of machine learning classifiers as well as MLP based neural network experiments. The MLP along with other traditional machine learning classifiers are based on a feature selection criterion. Therefore, we have considered a vast variety of features of which the best features were selected using the Chi-Squared method as explained in Section *Feature Extraction*. The proposed approach utilizes the features of both static and dynamic aspects of Android applications (for both the malware and benign applications on two platforms i.e., *real Android device* and the *emulator*). To gauge the comparative performance and detection rate among real and emulator devices, four machine learning, and one deep learning classifiers are used. The ROC curve of Gradient Boosting, KNN, RF, XGBoost with Neural Network under a real device can be seen in Fig. 3 and the ROC curve under a controlled environment is depicted in Fig. 4. Indicating a slight difference in the curve that shows somehow better results (conducted on a real Android device) as compared to the experiments conducted on the emulator device. These results indicate that Random Forest outperforms other models followed by the XGBoost and Neural Network (MLP). We experimented with perceptron-based neural networks. The model we present here has twenty hidden layers, each using the popular rectified linear unit (relu) activation function. The hyperparameters used for these MLP experiments are given in Table 5.

**Table 5:** MLP model hyperparameters.

Classifier	Device	Hyperparameters	Tested values
MLP	MLP	Hidden_layer_sizes	[20,20,20]
		Activation	[relu]
	Real + emulator	Solver	[adam]
		Max_iter	[100]

**Figure 5:**
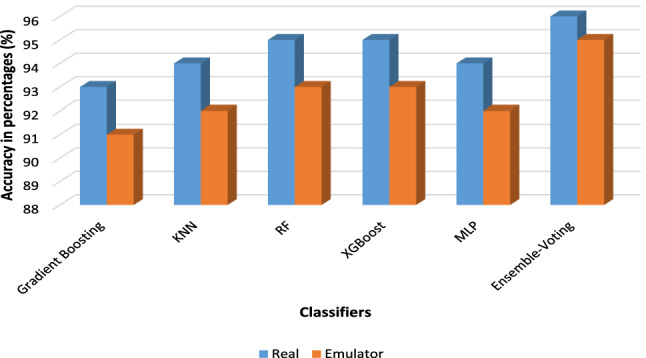
Accuracy comparing Neural Network with others under emulator and real device dataset.

**Table 6:** Accuracy, precision, recall and F1 score.

Device	Classifier	Accuracy (%)	Precision (%)	Recall (%)	F1 score (%)
Emulator device dataset	Gradient boosting	91	90	92	91
	KNN	92	91	94	92
	RF	93	92	95	94
	XGBoost	93	92	95	94
	MLP	92	91	94	92
Real device dataset	Gradient boosting	93	93	94	93
	KNN	94	94	95	94
	RF	95	95	96	95
	XGBoost	95	94	96	95
	MLP	94	93	96	94

**Table 7:** Summarizing the proposed approach with the state-of-the-art approaches in terms of platform, classifier, dataset used along with their accuracy and obfuscation technique applied.

Approaches	Device	Classifier	Dataset + Timeframe	Accuracy	Obfuscation	Ensemble learning
Suarez-Tangi et al. approach^2^	Emulator	Extra Tree	Malgenome 2011–2012 Drebin 2010–2012	99 %	Static-based obfuscation	–
Sihag et al. approach^21^	Emulator	k-NN J48 RF SMO	Androtopsy 2015 AndroTracker 2015 Drebin 2010–2012 PRAGuard 2015	92.47 %	Static-based obfuscation	
Lee at al.approach^22^	Emulator	CNN and RNN	VirusTotal 2014–2020	98.47%	Static-based obfuscation	–
Wu et al. approach^23^	Emulator	CNN	MalGenome 2011–2012 AndroZoo 2008–2020	98.4 %	Static-based obfuscation	–
Tang et al. approach^24^	Emulator	deep learning	MGOPDroid timeframe not mentioned	95.31 % without obfuscation 89.96% with obfuscation	Static-based obfuscation	–
Arp et al. approach^19^	Emulator	SVM	Drebin 2010–2012	93.90%	API reflection obfuscation	–
Garcia et al. approach^36^	Emulator	SVM CART	AndroZoo 2008–2020, MalGenome 2011–2012 Drebin 2010–2012, VirusShare 2008–2020, VirusTotal 2014–2020	85%	Four obfuscation	–
Proposed approach	Emulator & Real	Gradient Boosting KNN RF XGBoost Neural Network (MLP)	Kronodroid 2008–2020	93% on emulator 94% on real without obfuscation 91% on emulator as well as on real with obfuscation	Static + dynamic obfuscation	95.8% with obfuscation on real with ensemble

Furthermore, the performance and accuracy of real devices are better than the emulator-based experiments (as shown in Table 6 and Fig. 5). As a result, the comparative inspection of the two final datasets shows that the dynamic analysis of malware apps in the emulator is a more challenging task than in the real device. Whereas, the possibilities of conducting experiments in a real environment are not usually feasible. In addition, it can be seen from Table 6 that the Random Forest classifier outperforms the XGBoost in both environments by attaining 93% accuracy under a controlled environment whereas it attains 95% accuracy under the real environment without applying feature-based obfuscation technique. Moreover, an ensemble-voting mechanism based on a majority voting mechanism performs better (as shown in Fig. 5) which indicates that the veto-voting can improve the accuracy. Furthermore, summary of the state-of-the-art techniques with the proposed approach in terms of device used, classifiers implementation, dataset used, obfuscation techniques applied and learning mechanism usage are demonstrated in Table 7. Moreover, Table 7 is presented to highlight the shortcomings of the existing approaches with the proposed approach. It can be seen from Table 7, that most of the approaches used an outdated dataset and have shown significant classification results (considering only the basic malware apps classified using static aspect only). Whereas, sophisticated malware usually shows dynamic-based obfuscation which is investigated in this study by considering updated and most recent malware samples from Kronodroid dataset. Furthermore, a deep learning classifier is used after selecting the most significant features that had an impact on obfuscating applications. When considering deep learning classifier, the average processing time taken by the algorithm after providing an input to the proposed framework, it takes 0.91 seconds to compute application (server machine configured with Intel Core i7, 2.4GHz 6-Core with 16GB 2400 MHz DDR4 memory, Intel UHD graphics 630 1536 MB as depicted in Table 4). In addition, some of the approaches used a controlled environment (i.e., emulator) for experimentation, where most of the intelligent malware can easily hide their identity. However, we have used both emulator as well as real device platforms to observe and analyze the behavior of the advanced and obfuscated malware. Also, the samples taken from the datasets in the literature are relatively small, especially in the case of MalGenome which is composed of just 1260 samples whereas, the Drebin dataset consists of 5560 samples from the year 2010 to 2012. However, the dataset used in this study is a combination of old as well as recent malware from the year 2008 to 2020 consisting of 63,991 samples experimented in a controlled environment and 78,137 in a real Android platform.

To evaluate the effectiveness and to analyze the relative importance of the employed features for the detection and classification of malware, we have implemented a feature-based obfuscation mechanism to identify the vital attributes that play a significant role in the application obfuscation. For this purpose, we have applied a feature-obfuscation mechanism in such a way that reverting the values of a single category and comparing it with the other category (as shown in Fig. 2 and explained earlier in Section *Proposed Approach*). It can be seen that there is a small set of features that perform consistently well when they are derived from the basic malware application as shown in Table 6, however, after applying the feature-based obfuscation, a drastic change has been observed related to the relative importance of these features for obfuscating benign and malware applications. These results are presented in Table 8 where we can see that after applying feature-obfuscation in a controlled environment (i.e., an emulator), there seems slight variation in *intents* whereas no noticeable changes can be seen in the *others* category indicating the low involvement of these aspects in application obfuscation. Whereas, we can see that obfuscation plays a crucial role when feature-based-obfuscation is being applied on *permissions* and *system calls*. From Table 8, it can be seen i.e., a drastic change in accuracy, precision, recall, and F1 score. The significant change is the indication of the crucial role of these features i.e., if these features are obfuscated the detection rate could decrease significantly. Moreover, when feature-based-obfuscation is applied on real device, there is a slight change in accuracy with the intent-related features. A drastic decrease can be seen in system calls and permissions indicating the importance of these features in applying obfuscated mechanisms. Furthermore, it can be seen that when considering permissions and system calls features, MLP outperforms the other traditional ML approaches (showing the effectiveness of the method as compared to the basic ML methods).

Having less FN rate when compared to other classifiers where both FP and FN rates change (i.e., increases) in the same way. This indicates detection accuracy of both benign and obfuscated malware samples decreases in a similar way. The reason is that the benign applications are obfuscated (for example just to protect the intellectual property), and are mostly miss-classified. These results also show that the changes in permissions or system calls have a notable impact on the detection of malware too. Therefore, it is evident that the malware attackers obfuscate these features in a way to avoid detection, however, an in-depth analysis of these features can help in identifying the obfuscated pattern of Android device applications.

**Table 8:** Accuracy, Precision, Recall and F1 score after Feature-obfuscation.

Device	Feature-obfuscated	Classifier	Accuracy (%)	Precision (%)	Recall (%)	F1 score (%)
Emulator	Permissions	Gradient Boosting	75	75	76	75
		KNN	73	72	74	73
		RF	72	72	72	71
		XGBoost	73	72	75	74
		MLP	76	71	76	72
	Intents	Gradient Boosting	89	89	90	90
		KNN	89	89	91	90
		RF	91	90	91	90
		XGBoost	90	89	91	90
		MLP	90	89	91	90
	Others	Gradient Boosting	91	90	92	91
		KNN	91	90	92	91
		RF	91	90	92	91
		XGBoost	91	90	92	91
		MLP	91	90	92	91
	System Calls	Gradient Boosting	69	69	68	69
		KNN	69	69	69	69
		RF	72	73	72	71
		XGBoost	71	70	72	71
		MLP	71	70	72	71
Real	Permissions	Gradient Boosting	65	65	65	65
		KNN	68	68	69	69
		RF	70	71	70	71
		XGBoost	70	72	71	72
		MLP	71	71	72	72
	Intents	Gradient Boosting	91	90	92	91
		KNN	90	90	92	91
		RF	89	88	89	89
		XGBoost	88	87	88	88
		MLP	87	88	88	87
	Others	Gradient Boosting	89	89	89	89
		KNN	90	90	90	91
		RF	85	85	82	86
		XGBoost	86	86	85	86
		MLP	85	84	85	84
	System Calls	Gradient Boosting	68	68	67	68
		KNN	65	66	68	67
		RF	70	69	70	68
		XGBoost	61	60	62	61
		MLP	72	68	72	66

## Conclusion and future work

For regular smartphone users, Android malware poses a severe security danger. The obfuscation approach, however, can produce malware versions that can evade current detection strategies and dramatically lower detection accuracy. Attempting to identify Android malware obfuscation variations, in this paper, we proposed an approach to address the issues related to the classification and detection of Android malicious and obfuscated variants. In addition, we show that after applying a feature-based obfuscation technique, we get the most relevant features that play a vital role in detecting malware. Moreover, we demonstrated that a small subset of features, whether derived from plain or obfuscated malware, could potentially be used for a significant detection rate. The proposed deep-learning-based approach is a fast, scalable, and accurate mechanism for the detection of obfuscated Android malware. The designed model of the proposed approach comes up with a novel feature-based-obfuscated mechanism using chi-square as a selection algorithm. The experiments show that the proposed model detects malware accurately and effectively. Also, it identifies features that are usually obfuscated by malware attackers. This is crucial in situations where security analysts need intelligent tools to automatically detect and do additional analysis of malware that has been obfuscated. Our proposed model showed that the hybrid analysis for Android applications can be effective and aid in locating the obfuscated malware in a more suited manner. Future directions of this research include analysis of the packed Android applications in combination with the applying different types of obfuscations considering the least significant features too, whereas the analysis of such applications is a notable challenge.

## Data Availability

The data will be available as per reasonable request upon sending an email to an author: sanaaurangzeb@numl.edu.pk.
